# Classical Proprioceptors and Palisade Endings Have Distinct Molecular Profiles in Pig Eye Muscles

**DOI:** 10.1167/iovs.67.3.1

**Published:** 2026-03-02

**Authors:** Génova Carrero-Rojas, Arzu Petersen, Erdem Yildiz, Paula M. Calvo, Rosendo G. Hernández, Johannes Streicher, Rosa R. de la Cruz, Angel M. Pastor, Roland Blumer

**Affiliations:** 1 Center of Anatomy and Cell Biology, MIC, Medical University Vienna, Vienna, Austria; 2 Departamento de Fisiología, Facultad de Biología, Universidad de Sevilla, Sevilla, Spain; 3 Christian Doppler Laboratory for Inner Ear Research, Department of Otorhinolaryngology, Division of Head and Neck Surgery, Vienna General Hospital, Medical University of Vienna, Vienna, Austria; 4 Department of Anatomy and Biomechanics, Division of Anatomy and Developmental Biology, Karl Landsteiner University of Health Science, Krems an der Donau, Austria

**Keywords:** extraocular muscles (EOMs), proprioception, muscle spindles, Golgi tendon organs, palisade endings

## Abstract

**Purpose:**

Classical proprioceptors, such as muscle spindles and Golgi tendon organs, are absent from the extraocular muscles (EOMs) of most mammals, whereas palisade endings are present in most mammalian EOMs. Even-toed ungulates such as pigs are the only species with classical proprioceptors together with palisade endings in their EOMs. This study aimed to compare the molecular phenotype of classical proprioceptors and palisade endings in pig EOMs.

**Methods:**

EOMs from both eyes of nine pigs were analyzed. EOM cross sections and EOM whole mount preparations were immunolabeled with antibodies against neurofilament, glucose transporter 1 (GLUT1), choline acetyltransferase (ChAT), synaptophysin, synaptobrevin, complexin, and vesicular glutamate transporter 1 (VGLUT1). These were used together with the toxins phalloidin and α-bungarotoxin. The analyses were conducted using confocal laser scanning microscopy.

**Results:**

A capsule, expressing GLUT1, was present in muscle spindles and Golgi tendon organs but not in palisade endings. Conversely, palisade endings express ChAT, whereas muscle spindles and Golgi tendon organs lack it. An exception was the paraequatorial and polar regions of muscle spindles, where ChAT immunoreactivity was present. VGLUT1 was present in Golgi tendon organs and muscle spindles but absent from palisade endings. In classical proprioceptors as well as palisade endings, nerve terminals exhibited synaptophysin, synaptobrevin, and complexin immunoreactivity. Alpha-bungarotoxin was present in the motor terminal of the muscle spindle's paraequatorial and polar regions.

**Conclusions:**

Palisade endings and classical proprioceptors in pig EOMs differ regarding the capsular envelope and neurotransmitters. However, they equate each other with respect to proteins implicated in neurotransmitter release.

The ability to perceive the position and movement of body parts relies on proprioception, a sense mediated by specialized receptors known as proprioceptors. These receptors are distributed throughout skeletal muscles, skin, joints, and tendons. Typically, two proprioceptor organs - muscle spindles and Golgi tendon organs - are present in mammalian skeletal muscle.^1^^–^^3^ They provide continuous central feedback on muscle length and tension, enabling balance maintenance, postural adjustments, and coordinated movements.^1^^,^^4^

The eyes are the most mobile organs of the mammalian body, and vision is only useful when the direction of gaze is known. Consequently, it was hypothesized that the extraocular muscles (EOMs) contain proprioceptors.^5^ However, the opposite is the case, and only even-toed ungulates, such as cattle, pigs, and sheep, have muscle spindles and Golgi tendon organs in their EOMs, where they occur in high numbers and closely resemble those found in skeletal muscles.^6^^–^^8^ In primate EOMs, muscle spindles are found in humans^9^^,^^10^ and monkeys,^11^ whereas Golgi tendon organs are only found in monkeys.^12^ However, it is important to note that human EOM muscle spindles exhibit a simplified structure, which is even more simplified in monkeys.^9^^–^^11^^,^^13^ The rest of the species lack both muscle spindles and Golgi tendon organs in the EOMs.^7^ The evolutionary and functional significance of why only a few species have proprioceptors in their EOMs remains to be elucidated.

Despite the absence of proprioceptors in most mammals, the brain receives eye position information.^14^^–^^16^ This paradox has prompted the search for alternative proprioceptors in EOMs. One such candidate is the palisade ending, a specialized nerve end organ found in the EOMs of most mammals. Located at the myotendinous junction, palisade endings have been proposed as the source of eye position signals.^17^^–^^21^ However, their cholinergic nature and the location of their cell bodies within oculomotor nuclei have put the sensory role of palisade endings into question.^20^^,^^22^^–^^24^ The function of palisade endings remains a subject of ongoing debate.

Even-toed ungulates are the only species that possess classical proprioceptors and palisade endings in their EOMs. In the light of the ongoing debate surrounding the function of palisade endings, specifically the question of whether they serve sensory or motor functions, we performed a comparative analysis of the molecular composition of classical proprioceptors and palisade endings. Key molecules of classical proprioceptors in skeletal muscles are glucose transporter 1 (GLUT1)^25^ and vesicular glutamate transporter 1 (VGLUT1).^3^^,^^4^^,^^26^ Core molecules in palisade endings are choline acetyl transferase (ChAT), synaptophysin, synaptobrevin, and complexin.^22^^,^^27^ In the present study, we tested the expression of these molecules in classical proprioceptors and palisade endings of pig EOMs. We show that palisade endings and classical proprioceptors differ regarding GLUT1, VGLUT1, and ChAT expressions. However, they equal each other with respect to synaptophysin, synaptobrevin, and complexin expression.

## Materials and Methods

### Animals

A total of nine adult pigs were analyzed in the present study. Six pigs were from the Animal facility of Cordoba University (Spain) and three pigs were from the Core Facility Laboratory Animal Breeding and Husbandry (CFL) of the Medical University of Vienna (Austria). All procedures were conducted in compliance with the National Institutes of Health (NIH; http://oacu.od.nih.gov/) guidelines, specifically those for the care and maintenance of higher mammals in neuroscience experiments (NIH publication #94-3207, 1994). In addition, the study adhered to Spanish legislation on the use and care of laboratory animals (R.D. 53/2013, BOE 34/11370-421, 2013). The animal study was reviewed and approved by the Austrian Federal Ministry for Science and Research (BMBWF-2023-0.455.997).

### Tissue Preparation

Pigs were killed with a terminal dose of sodium pentobarbital (100 mg/kg, intraperitoneally [IP]). Subsequently, the eyeballs, including the EOMs, were carefully dissected. Four rectus muscles (superior, inferior, lateral, and medial rectus muscles) from both eyes were collected from each animal. Muscles were fixed in 4% paraformaldehyde in phosphate buffered saline (PBS) for 2 hours. The EOMs were then cut transversely into two parts: one containing the muscle belly and the other comprising the distal third of the muscle with the attached tendon. The distal EOM portions were prepared as whole mount preparations. The samples were subjected to immediate processing or stored at 4°C in sodium PBS containing 0.05% sodium azide to prevent bacterial contamination, prior to further processing. The muscle bellies were further processed for sectioning.

Muscle bellies were cryoprotected in graded sucrose solutions (10%, 25%, and 40%) prepared in PBS, followed by a final step at 40% sucrose with Cryomatrix (Thermo Fisher Scientific, Waltham, MA, USA) in a 1:1 ratio for 24 hours. Subsequently, the samples were embedded in Cryomatrix, frozen in ice-cooled 2-methylbutane, and stored at −80°C. Cryosections were cut at either 10 µm (for cross-sections of the muscle belly) or 200 µm thickness (for whole mount preparations of the muscle belly). The distal EOM portions and cryosections underwent multicolor immunofluorescence staining.

### Antibodies and Toxins

The following antibodies and toxins were used: GLUT 1 (GLUT1) to label capsular cells^25^; anti-neurofilament as a pan-axonal marker^28^; anti-synaptophysin and anti-synaptobrevin for presynaptic vesicles^29^^,^^30^; anti-complexin, a marker for a cytoplasmic protein that promotes neurotransmitter release^31^; anti-choline acetyltransferase (ChAT) as a marker of cholinergic axons^20^^,^^32^; and anti-VGLUT 1 (VGLUT1) to identify glutamatergic synapses.^33^ The toxin phalloidin was used to label muscle fibers^34^ and the snake venom α-bungarotoxin was used to label motor endplates.^35^ The primary antibodies and toxins, along with their working dilutions, RRID numbers, and suppliers, are detailed in the Table. Secondary antibodies were obtained from Thermo Fisher Scientific (Waltham, MA, USA). These were raised in either goat or rabbit and conjugated with Alexa Fluor 568 or Alexa Fluor 488 and used at a dilution of 1:500. Phalloidin was conjugated with AF647, and α-bungarotoxin was conjugated with AF647 or rhodamine.

**Table.** List of Markers Used in the Present Study, Including Working Dilution, RRID Number, and Suppliers

Primary Antibodies and Toxins			
Markers	Working Solution	Catalog/RRID Number	Supplier
Antibodies			
Anti-neurofilament	1:2000	AB5539/AB_177520	Merck/Millipore
Anti-glucose transporter 1	1:500	AB115730/AB115730	ABCAM
Anti-choline acetyltransferase	1:100	AB144P/AB_2079751	Merck/Millipore
Anti-synaptophysin	1:300	MAB329/AB_95786	Merck/Millipore
Anti-synaptobrevin	1:100	MAB335/AB_11213070	Merck/Millipore
Anti-complexin	1:500	122003/AB_2619793	Synaptic System
Anti-vesicular glutamate transporter 1	1:500	135303/AB_887875	Synaptic System
Toxins			
Phalloidin	1:200	65906-10NMOL	Sigma-Aldrich
α-bungarotoxin	1:500	B35450	Thermo Fisher

### Immunofluorescence

A series of multicolor immunofluorescence staining experiments were performed to define selective patterns of expression of markers in afferent and efferent axons of the extraocular muscles. This staining strategy enabled the differentiation of GLUT1-positive versus GLUT1-negative capsule cells, cholinergic versus non-cholinergic nerve fibers, sensory versus motor nerve terminals, and VGLUT1-positive versus VGLUT1-negative nerve terminals. The staining protocols utilized for both sections and whole mount preparations varied and are delineated below.

#### Immunofluorescence Protocol for Cryo-Sections

Defrosted sections were rinsed in PBS and blocked for 1 hour with 10% goat or rabbit serum in PBS containing 0.1% Triton X-100 (PBS-T). After blocking, the sections were incubated with the primary antibodies diluted in PBS-T 0.1% for 24 hours at 4°C. Following thorough washing in PBS, the sections were incubated for 2 hours with secondary antibodies and/or phalloidin or α-bungarotoxin. Finally, the sections were washed with PBS and coverslipped with an aqueous mounting medium (Dako, Agilent, Santa Clara, CA, USA) for subsequent microscopy analysis.

#### Immunofluorescence Protocol for Whole Mount Preparations

Immunolabeled whole mount preparations allowed us to visualize the entire structure of muscle spindles, Golgi tendon organs, and palisade endings. Before immunolabeling, the tissue samples were frozen in ice-cooled 2-methylbutane at −80°C and then rinsed in PBS-T 1% for 24 hours. After this, they were blocked for 1.5 hours with 10% goat or rabbit serum diluted in PBS-T 1%, which was then followed by incubation with primary antibodies diluted in PBS-T 1% for 48 hours at room temperature. After thorough washing in PBS-T 1%, samples were incubated for 6 hours at room temperature with secondary antibodies and/or either phalloidin or α-bungarotoxin. Finally, the muscles were rinsed in PBS-T 1% and mounted in concave cavity slides with a 60% glycerol/40% PBS solution for subsequent analysis under the microscope.

### Immunofluorescence Data Analysis

Fluorescently labeled cross-sections and whole mount preparations were examined using a confocal laser scanning microscope (CLSM; Olympus FV3000, Olympus Europa SE & Co. KG, Hamburg, Germany). A series of virtual CLSM sections, each 1-µm thick, was obtained through the structure of interest. Each section was digitally acquired at a resolution of 1024 × 1024 pixels, and three-dimensional projections were generated using ImageJ software (NIH, Bethesda, MD, USA). Images were acquired using lasers with excitation wavelengths of 488, 568, and 633 nm. In some cases, bright-field images were generated using an excitation wavelength of 647. Bright field images and colored images were combined. This technique was used to correlate immunolabeled structures with the corresponding morphology.

## Results

### Structure and Molecular Features of Muscle Spindles

**Figure 1.**
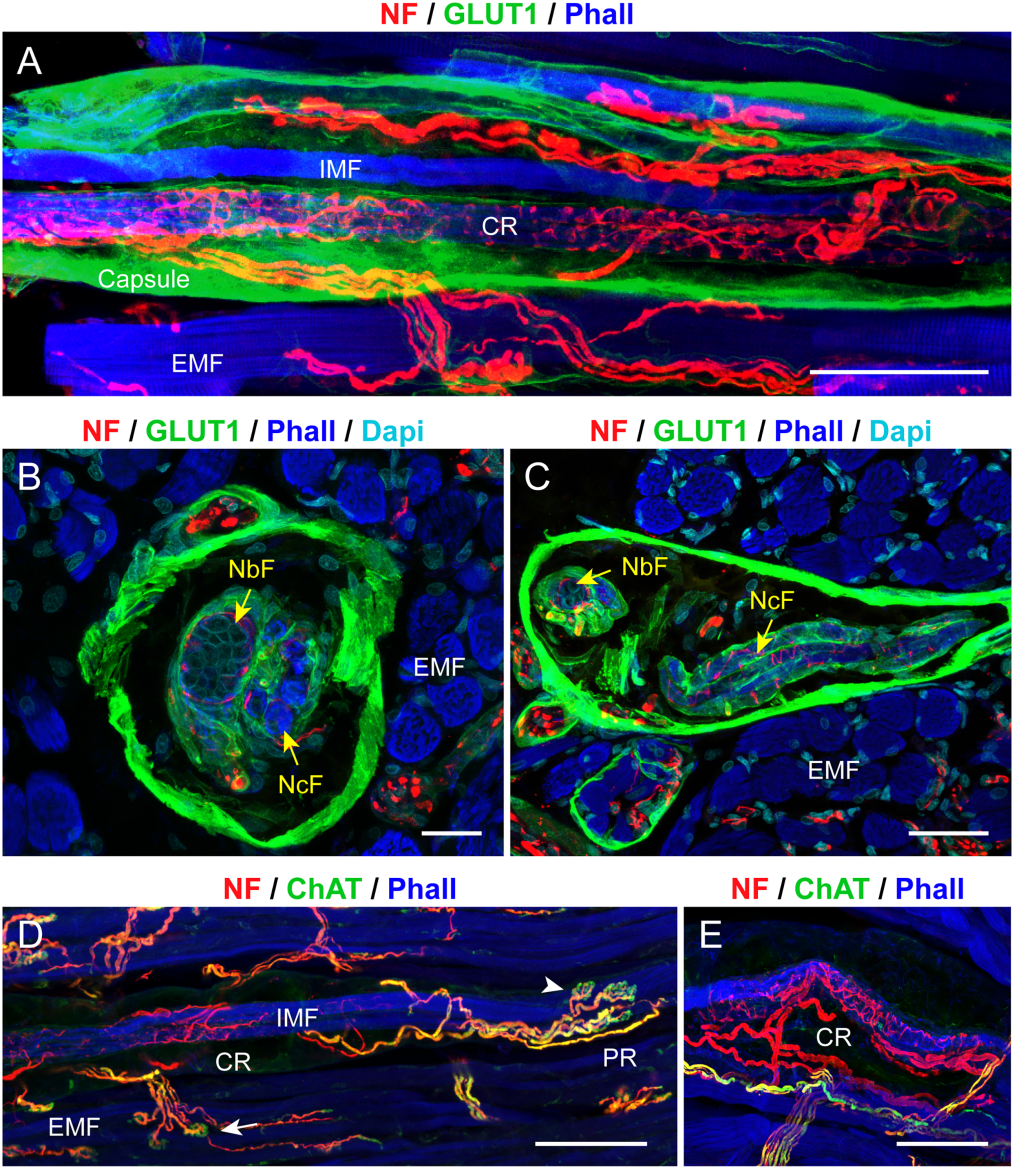
**Structural and molecular signature of muscle spindles in pig EOMs.** (**A–C**) Labeling with anti-neurofilament (NF; *red*), anti-glucose transporter 1 (GLUT1; *green*), in a whole mount preparation in **A** and cross-sections in **B** and **C**. In these and other images, muscle fibers are labeled with phalloidin (Phall; *blue*). Muscle spindles have a capsule that expresses GLUT1 in **A** to **C**. Inside the spindle, intrafusal muscle fibers (IMFs in **A**), comprising nuclear chain fibers (NcFs in **B** and **C**) and nuclear bag fibers (NbFs in **B** and **C**) are present. Nuclear chain and NbFs are surrounded by cells expressing GLUT1. Several nerve fibers enter the muscle spindles in the central region (CR), and upon entering, wrap around the intrafusal muscle fibers in **A**. (**D**, **E**) Whole mounts labeled with anti-NF (*red*), anti-ChAT (*green*), and phalloidin. Nerve fibers wrapping around intrafusal muscle fibers (IFMs) in the central region (CR) are ChAT-negative **E**. ChAT-positive fibers establish motor terminals on intrafusal fibers in the polar regions of the muscle spindle (*white arrowhead* in **D**). Extrafusal muscle fiber (EMF) with a motor terminal (*white arrow* in **D**). *Scale bars* = 100 µm in **A**; 25 µm in **B**; 50 µm in **C**; and 100 µm in **D** and **E**.

Muscle spindles were in the outer layer (orbital layer) of the EOMs. They had a fusiform shape and a capsule that expressed GLUT1 (Figs. 1A–C). The mean diameter of the muscle spindles in the central region was 103.9 ± 16.5 µm (mean ± SD). Inside the capsule between 2 and 18 (mean 9.9 ± 2.6) intrafusal fibers were present. They were encircled by an epimysial capsule that exhibited GLUT1 immunoreactivity (see Figs. 1B, 1C). Axons likely providing innervation to the muscle spindle also exhibited GLUT1 perineurial capsule. Thus, GLUT1 formed an endomysial-perimysial sheath encapsulating the spindle muscle fibers that also extended to the innervating bundles of axons as a perineurial sheath. Two categories of intrafusal fibers according to the nuclear arrangement in the central region of the fiber could be distinguished: nuclear chain and nuclear bag fibers, with the former exhibiting a string of central nuclei, and the latter exhibiting a focal accumulation of nuclei (see Figs. 1B, 1C). In every muscle spindle, the nuclear chain fibers significantly exceeded the nuclear bag fibers, with mean numbers of 8.3 ± 1.8 and 1.2 ± 0.9, respectively.

**Figure 2.**
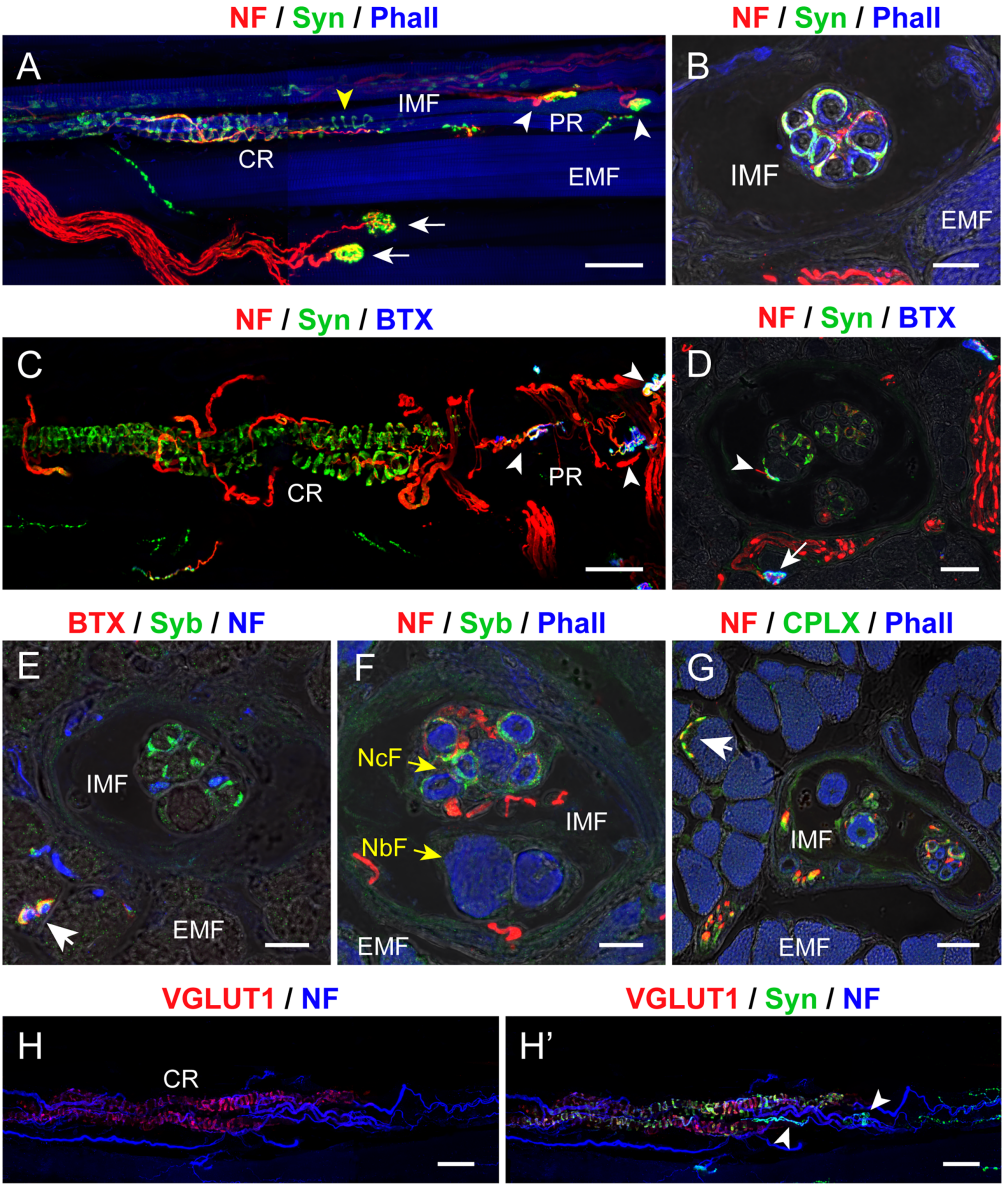
**Presynaptic markers in muscle spindles from pig EOMs.** (**A–D**) Labeling with anti-neurofilament (NF; *red*) and with anti-synaptophysin (Syn; *green*) along with phalloidin (Phall; *blue* in **A** and **B**) or α-bungarotoxin (BTX; *blue* in **C** and **D**) in whole mount preparations **A** and **C** and cross-sections **B** and **D**. Annulospiral sensory endings (*yellow arrowhead*) in the central region (CR) and motor terminals (*white arrowheads*) in the spindle's paraequatorial and polar region (PR) express synaptophysin in **A**, **B**, **C**, and **D**. Motor terminals, contacting intrafusal muscle fibers (IMFs) and extrafusal muscle fibers (EMFs), express synaptophysin and α-bungarotoxin (*white arrowheads* in **C** and **D**, *white arrow* in **D**). The color combination of *red*, *green*, and *blue* along with the varying color intensity, results in *white* or *cyan*-colored motor terminals. The images in **C** and **D** are illustrated in split channels in the Supplementary Figures S1A, S1A’, S1B and S1B”. (**E**, **F**) Cross-sections through the muscle spindle's central region labeled with anti-NF (*blue* in **E**; NF, *red* in **F**) and anti-synaptobrevin (Syb; *green*) along with α-bungarotoxin (BTX; *red*) in **E** and phalloidin (Phall; *blue*) in **F**. Annulospiral nerve terminals express synaptobrevin **E** and **F**. A motor terminal (*white arrow* in **E**) outside the muscle spindle expresses synaptobrevin and α-bungarotoxin. Nuclear chain fibers (NcFs; *yellow arrow*) and nuclear bag fibers (NbFs; *yellow arrow*). Intrafusal muscle fibers (IMFs). (**G**) Cross-section through the muscle spindle's central region labeled with anti-NF (*red*), anti-complexin (CPLX; *green*), and phalloidin (Phall; *blue*). Complexin is expressed in nerve fibers as well as annulospiral nerve terminals. A motor terminal (*white arrow* in **G**) outside the muscle spindle expresses complexin. (**H,** **H’**) Labeling with anti-NF (blue), anti-VGLUT1 (*red*), and anti-synaptophysin (Syn, *green*). Annulospiral endings in the spindle's central region express VGLUT1 and synaptophysin. Motor terminals (*arrowheads*) lack VGLUT1. *Scale bars* = 50 µm in **A** and **C**; 15 µm in **B**, **D**, **E**, and **F**; 25 µm in **G**; and 50 µm in **H** and **H’**.

At the level of the muscle spindle's central region several nerve fibers entered the muscle spindles. Most fibers were ChAT-positive, whereas a lower number was ChAT-negative (Figs. 1D, 1E). Upon entering, ChAT-negative nerve fibers spiraled around intrafusal fibers in the central region of the muscle spindle, thereby forming annulospiral sensory nerve endings (see Fig. 1E). Annulospiral nerve terminals were immunoreactive for synaptophysin (Figs. 2A–D), synaptobrevin (Figs. 2E, 2F), complexin (Fig. 2G), and VGLUT 1 (Figs. 2H, 2H’). Annulospiral nerve terminals lacked α-bungarotoxin (see Figs. 2C–E).

Motor terminals were found immediately adjacent to the muscle spindle's central region, in the paraequatorial region (see Figs. 2C, 2D) or at some distance away, in the muscle spindle's polar region (see Fig. 2C). The Figures 2C and 2D are displayed in split channels in the Supplementary Figures S1A, S1A’, and S1B and S1B”. Motor terminals were formed by ChAT-positive nerve fibers (see Fig. 1D) and exhibited α-bungarotoxin opposed to the terminal (see Figs. 2C, 2D). At the presynaptic site, they expressed synaptophysin (white arrowheads in Figs. 2A, 2C, 2D), synaptobrevin (Supplementary Figs. S1C, S1C”), and complexin (Supplementary Figs. S1D, S1D”), but not VGLUT1 (white arrowheads in Figs. 2H, 2H’).

### Structure and Molecular Features of Golgi Tendon Organs

**Figure 3.**
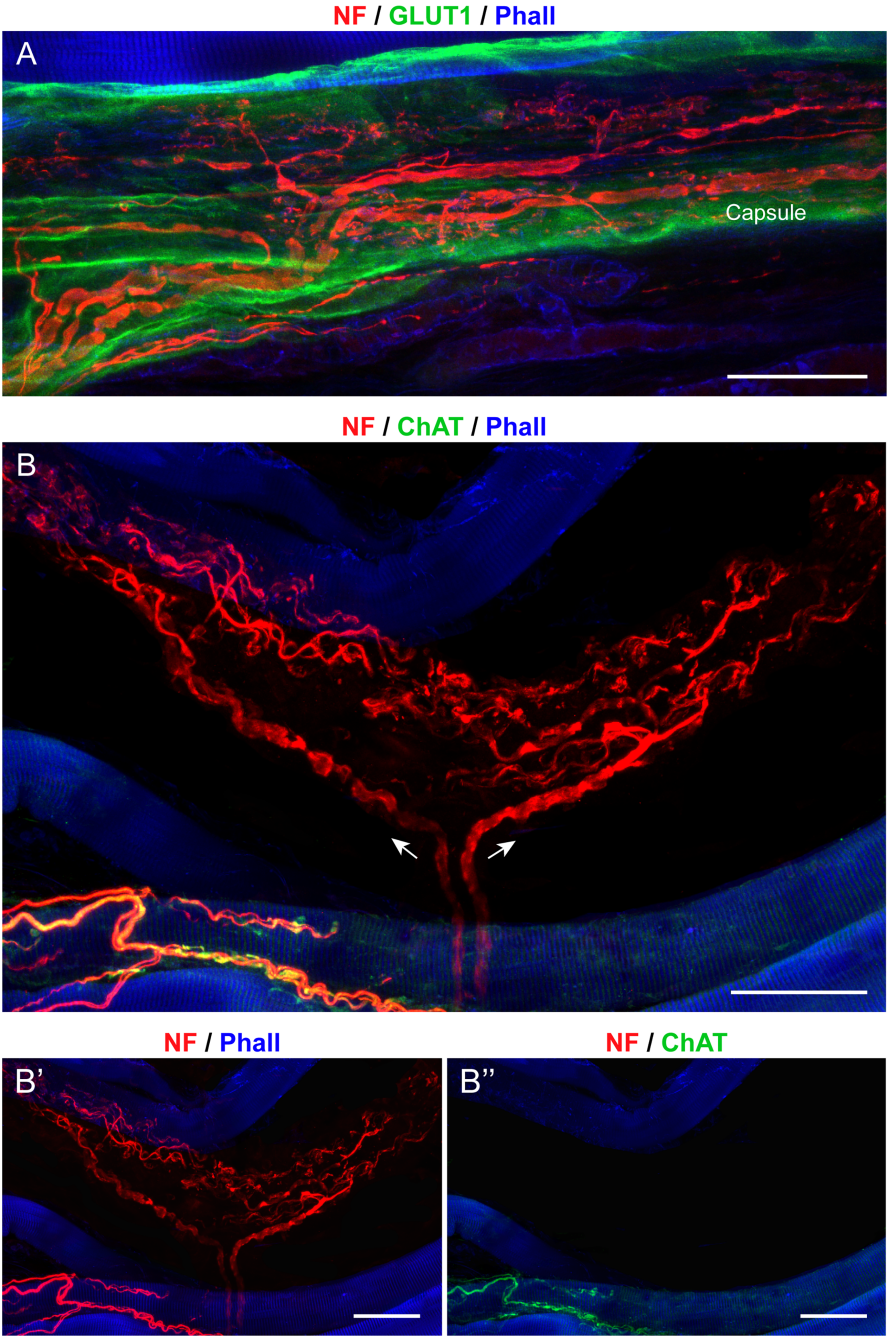
**Structural and molecular signature of Golgi tendon organs in pig EOMs.** (**A**, **B,** **B”**) Whole mount preparations labelled with neurofilament (NF; *red*) and phalloidin (Phall; *blue*), along with GLUT1 (*green*) in **A** and ChAT (*green*) in **B** and **B”**. The Golgi tendon organ has a capsule that expresses GLUT1 **A**. Nerve fibers entering the Golgi tendon organ (*white arrows* in **B**) are ChAT-negative (**B,** **B”**). Upon entering, nerve fibers extend in opposite directions and further divide into several thin branches in **A**, and **B**. *Scale bars* = 50 µm in **A**, **B**, and **B”**.

**Figure 4.**
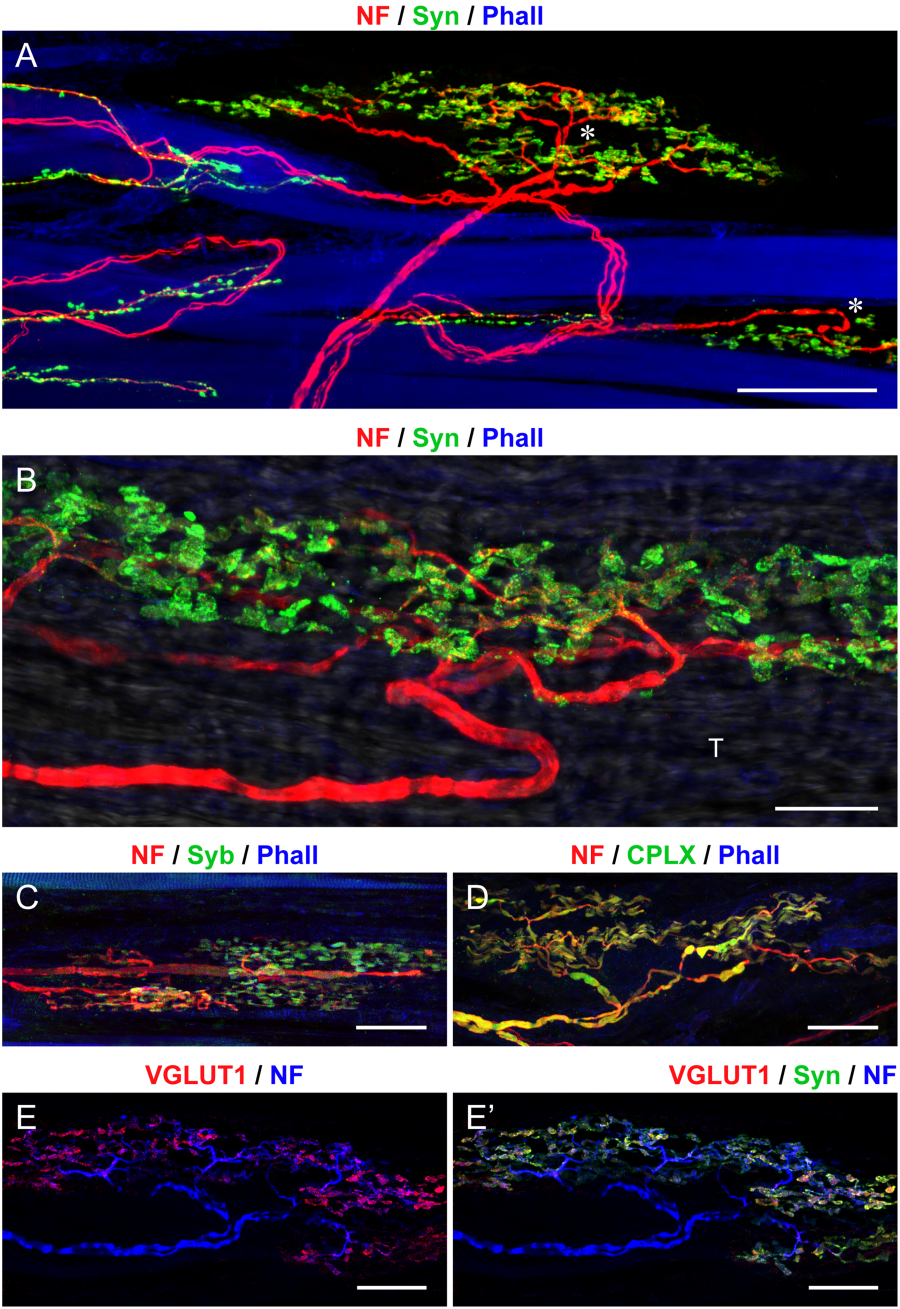
**Presynaptic markers in Golgi tendon organs from pig EOMs.** (**A,** **B**) Whole mount preparation labeled with anti-neurofilament (NF; *red*), anti-synaptophysin (Syn; *green*), and phalloidin (Phall; *blue*). Nerve fibers innervating Golgi tendon organs (*asterisks* in **A**) establish synaptophysin-positive terminals with the tendon bundles of the Golgi tendon organ in **A** and **B**. In **B**, the tendon (T) is visualized using the light transmission technique. (**C**, **D**) Whole mount preparation labelled with anti-NF (*red*) along with anti-Syb (*green*), and Phall (*blue*) in **C** and with anti-complexin (CPLX; *green*), and Phall (*blue*) in **D**. Nerve terminals in the Golgi tendon organ express synaptobrevin in **C**. Complexin is expressed in nerve fibers innervating Golgi tendon organs and in nerve terminals in **D**. (**E,** **E’**) Whole mount preparation labeled with NF (*blue*), VGLUT1 (*red*), and Syn (*green*). Axon terminals in the Golgi tendon organ express VGLUT1 in **E**, which colocalizes with synaptophysin in **E’**. *Scale bars* = 100 µm in **A**; 50 µm in **B**, **C**, **D**, **E,** and **E’**.

The Golgi tendon organs were located either at the myotendinous junction or far within the tendon. These organs had a fusiform shape and a capsule that exhibited GLUT1 expression (Fig. 3A). The Golgi tendon organs were innervated by a single large-diameter axon, which sometimes is divided into two or more main branches before entering the organ (Figs. 4A, 4B). Nerve fibers supplying Golgi tendon organs did not express ChAT (Figs. 3B, 3B”). Upon entering, the axon underwent bifurcation, with nerve branches extending in opposite directions (Fig. 3B). After further branching, nerve fibers established nerve terminals that did not express ChAT with the collagen bundles that filled the Golgi tendon organ (see Figs. 3B, 3B’’). In turn, Golgi tendon organ nerve terminals were immunoreactive for synaptophysin (see Figs. 4A, 4B), synaptobrevin (Fig. 4C), complexin (Fig. 4D), and VGLUT1 (Figs. 4E, 4E’).

### Structure and Molecular Features of Palisade Endings

**Figure 5.**
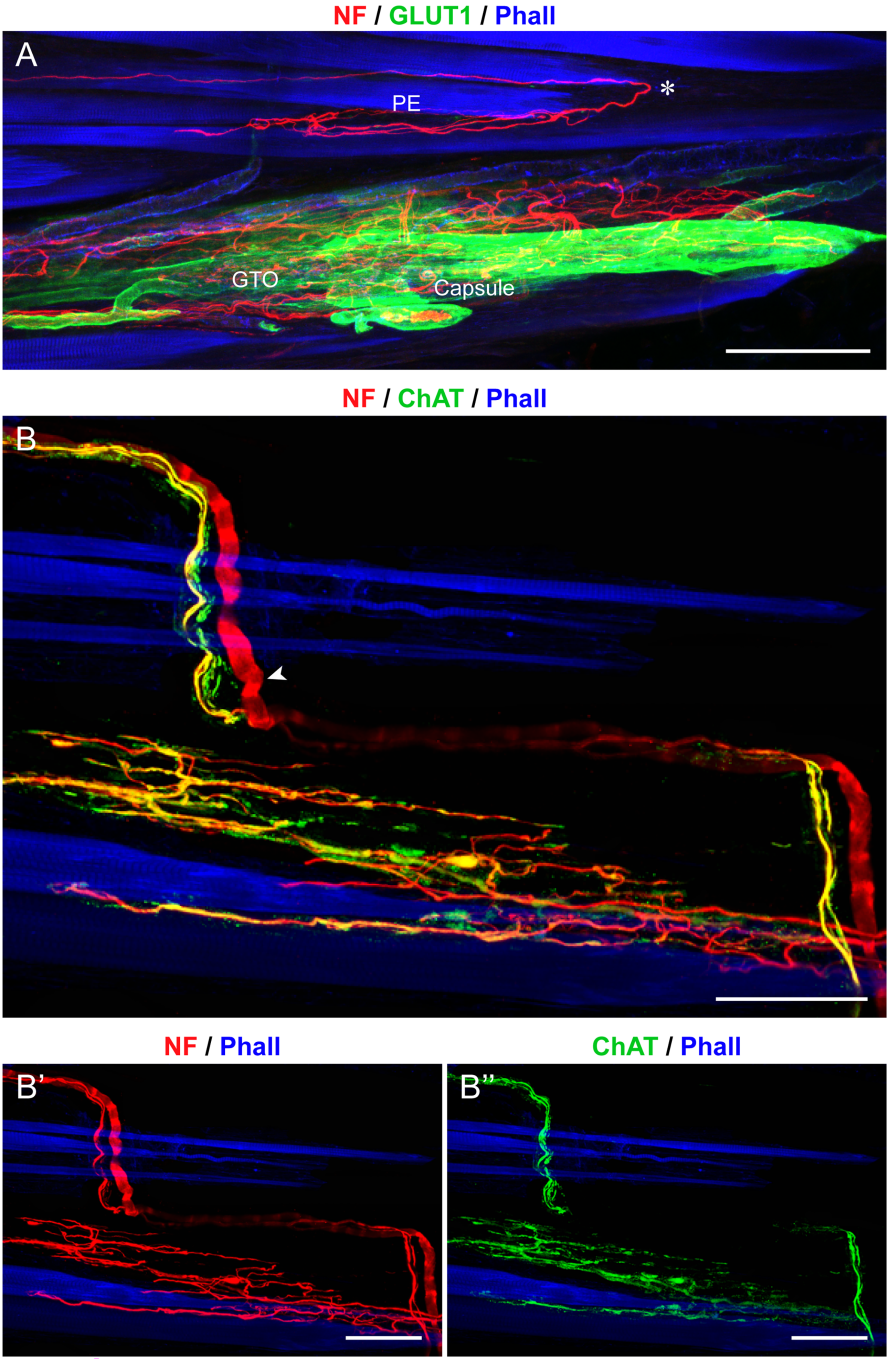
**Structural and molecular signature of palisade endings in pig EOMs.** (**A**) Whole mount preparation labeled with anti-neurofilament (NF; *red*), GLUT1 (*green*), and phalloidin (Phall; *blue*). The palisade ending (PE) does not express GLUT1, whereas the capsule of the Golgi tendon organ (GTO) does express GLUT1. The *asterisk* (*) indicates the point where the axon forming the PE turns back 180 degrees to approach the muscle fiber tip. (**B,** **B’’**) Whole mount labeled with anti- NF (*red*), anti-ChAT (*green*), and Phall (*blue*). NF-positive axon forming PE expresses ChAT in **B** and **B’’**. The thick ChAT-negative axon (*white arrowhead*) is indicative of a nerve fiber innervating a Golgi tendon organ. *Scale bars* = 100 µm in **A**; 50 µm in **B** and **B’’**.

**Figure 6.**
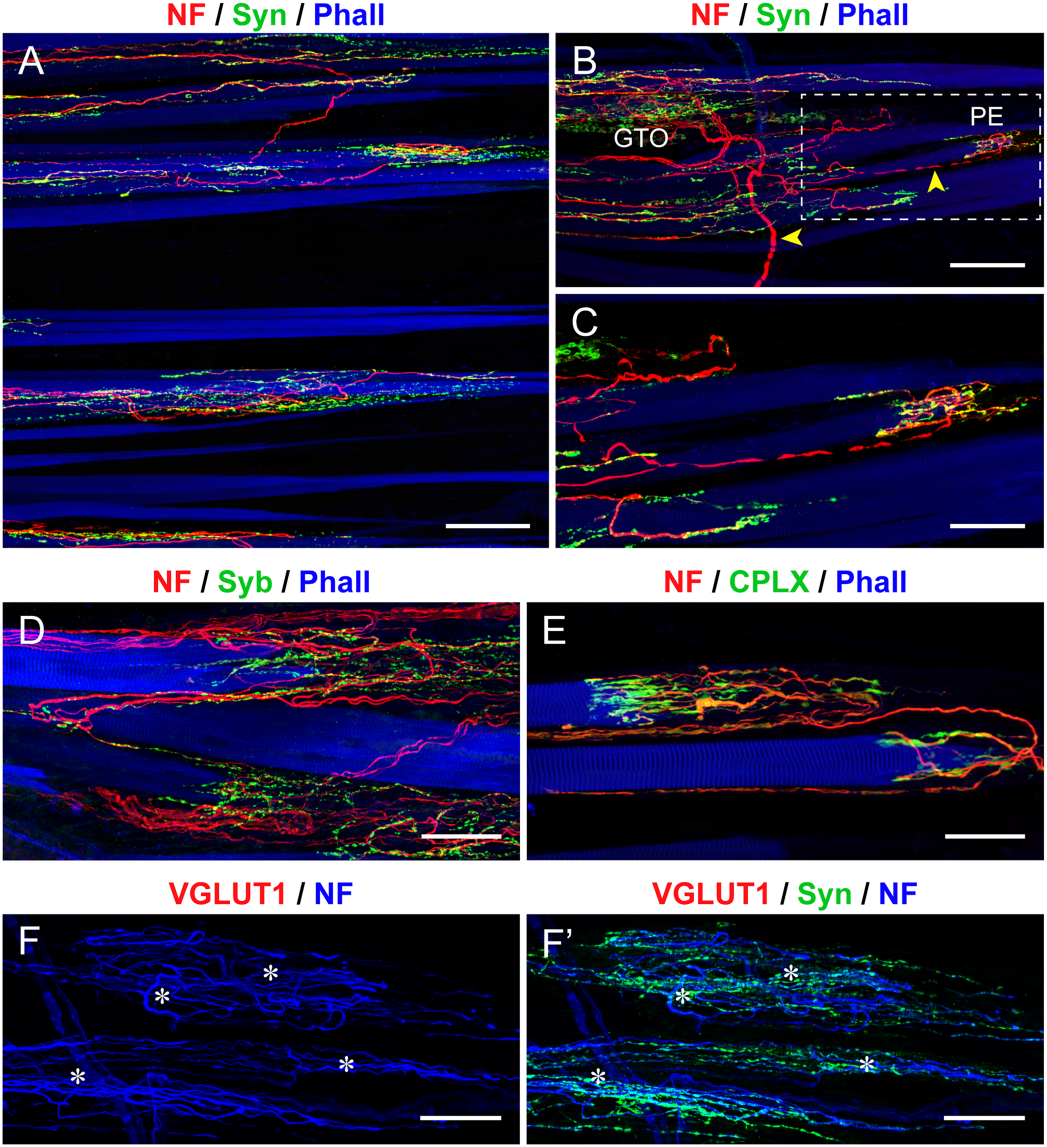
**Presynaptic markers in palisade endings from pig EOMs.** (**A–C**) Whole mount preparations labelled with anti-neurofilament (NF; *red*), anti-synaptophysin (Syn; *green*), and phalloidin (Phall; *blue*). Panoramic image in **A** and high magnification images in **B** and **C**, showing palisade endings (PEs) that express synaptophysin. A Golgi tendon organ (GTO) and a PE are present in **B**. The nerve fibers (*yellow arrowheads*), innervating the Golgi tendon organ and the PE have different thickness. The PE in **B** (*boxed inset*) is shown at higher magnification in **C**. (**D**, **E**) Whole mount labelled with anti-NF (*red*) along with anti-synaptobrevin (Syb; *green*), and phalloidin (Phall; *blue*) in **D**, and anti-complexin (CPLX; *green*) and phalloidin (Phall; *blue*) in **E**. PEs express synaptobrevin (**D**) and complexin (**E**). (**F,** **F’**) Whole mount labeled with anti- NF (*blue*), anti-VGLUT1 (*red*), and anti-synaptophysin (Syn; *green*), showing that PEs (*) do not express VGLUT1. *Scale bars* = 100 µm in **A** and **B**; 50 µm in **C**, **D**, **E**, **F,** and **F’**.

Palisade endings were found at the myotendinous junctions of the EOMs and did not express GLUT1 (see Fig. 5A). They originated from ChAT-positive nerve fibers that, arising from the muscle, entered the tendon (Figs. 5B, 5B”). Within the tendon, the nerve fibers made a 180-degree turn, followed by subsequent branching, resulting in the establishment of terminal varicosities around single muscle fiber tips. Palisade nerve terminals expressed ChAT (see Figs. 5B, 5B”), synaptophysin (Figs. 6A–C), synaptobrevin (Fig. 6D), and complexin (Fig. 6E). However, these organs lacked VGLUT1 immunoreactivity (Figs. 6F, 6F’).

## Discussion

**Figure 7.**
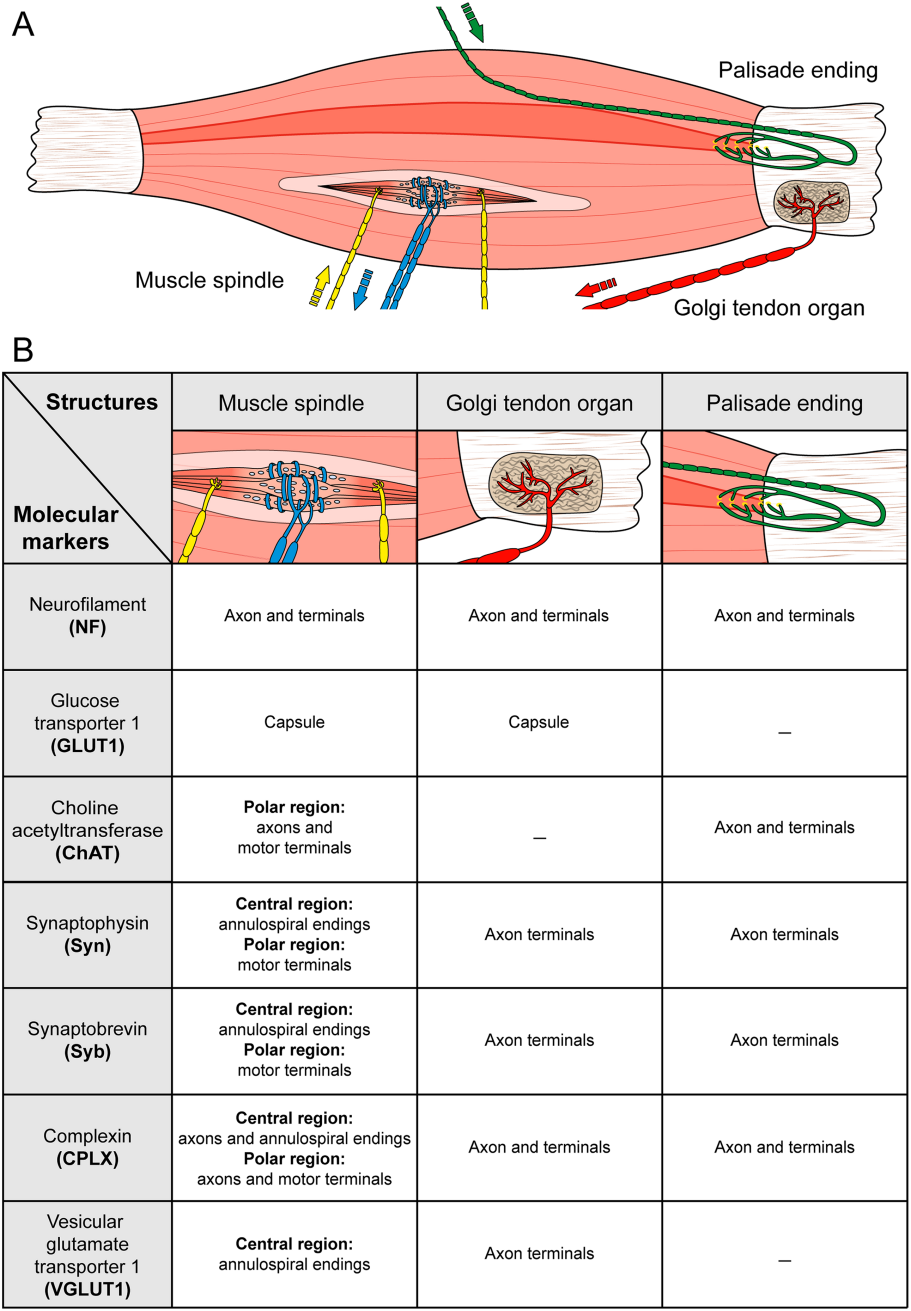
**Schematic diagram summarizing the main results of the present work.** (**A**) Illustration of a muscle spindle, a Golgi tendon organ, and a palisade ending in pig EOMs. The muscle spindle is located at the muscle belly. It contains intrafusal muscle fibers that are innervated by afferent sensory axons (*blue*) in the central region. Efferent axons (*yellow*) establish motor contacts in the muscle spindle's paraequatorial and polar regions. The Golgi tendon organ is located at the muscle-tendon junction and is filled with collagen bundles. A single large afferent axon (*red*) enters the Golgi tendon organ and splits into several branches. The palisade ending is also located at the muscle-tendon transition. It is formed by an efferent axon (*green*) that turns back 180 degrees and establishes synaptic contacts around a single muscle fiber tip. The *arrows* show the direction of the signal (afferent versus efferent). (**B**) Table summarizing the expression of the markers used in this study in muscle spindles, Golgi tendon organs, and palisade endings.

The classical proprioceptor pair, the muscle spindle and the Golgi tendon organ, is absent in the EOMs of most mammals, and, instead, palisade endings are present. Even-toed ungulates, such as pigs, are an exception because their EOMs contain both classical proprioceptors and palisade endings.^36^ These species are ideal models for conducting comparative analyses at the molecular level within the same animal to assess the potential of palisade endings to compensate for the absence of muscle spindles and Golgi tendon organs. In the present study, we show that classical proprioceptors and palisade endings exhibit distinct patterns of GLUT1, VGLUT1, and ChAT expressions. We also show that the expression of synaptophysin, synaptobrevin, and complexin is conserved in these organs. Figure 7 summarizes our findings.

In mammals with muscle spindles and Golgi tendon organs in their EOMs, the capsule of these organs is formed by perineurial cells.^8^^,^^37^ In this study, we demonstrate that the capsule cells of EOM proprioceptors express GLUT1. This is in line with a recent study,^25^ which showed that the capsule of muscle spindles and Golgi tendon organs in murine skeletal muscle express the perineurial marker GLUT1. Different from skeletal muscle spindles, GLUT1 expression was also found around individual intrafusal muscle fibers of the pig EOM spindles. This suggests the potential involvement of perineurial cells in the internal compartmentalization of these organs. The capsule of palisade endings is distinct from classical proprioceptors in that it is formed by fibrocytes.^8^^,^^21^^,^^23^ Furthermore, the present study shows that the capsule of palisade endings does not express GLUT1. These findings demonstrate that the ensheathment of classical proprioceptors and palisade endings exhibits distinct structural and molecular differences.

In skeletal muscle spindles, the capsule functions as a diffusion barrier, thereby protecting the annulospiral endings from the surrounding extracellular milieu.^38^ It is conceivable that the capsules of the EOM muscle spindles and EOM Golgi tendon organs fulfills an analogous function. Whether the capsule of palisade endings has a protective function is not known. In the event that this is the case, palisade endings rely on a non-perineurial form of protection.

Besides differences in the capsular envelopment, palisade endings and classical EOM proprioceptors of pig EOMs manifest neuromolecular differences. Specifically, palisade endings express ChAT, but not VGLUT1. To the contrary, muscle spindles and Golgi tendon organs do not express ChAT but do express VGLUT1. An exception is the paraequatorial and polar region of muscles spindles, where ChAT immunoreactivity is present presumably corresponding to gamma motor axons.

The presence of ChAT in palisade endings of pig EOMs is consistent with findings in other species.^23^^,^^24^^,^^27^ Furthermore, the absence of ChAT in EOM spindles, except for the muscle spindle's paraequatorial/polar region, and EOM Golgi tendon organs is in line with our previous study in sheep.^8^ ChAT is the synthesizing enzyme for neurotransmitter acetylcholine. Consequently, ChAT-positive palisade endings contain acetylcholine, whereas ChAT-negative muscle spindles (except the paraequatorial/polar region) and ChAT-negative Golgi tendon organs do not contain this neurotransmitter. The presence of ChAT suggests an effector role for palisade endings rather than a sensory role, which is also in agreement with their origin in the EOM motor nuclei.^20^^,^^24^ VGLUT1 expression has been demonstrated in muscle spindles and Golgi tendon organs of skeletal muscles.^33^^,^^39^ VGLUT1 is a vesicle membrane protein that transports glutamate into the synaptic vesicles of annulospiral nerve terminals of muscle spindles.^40^ Glutamate plays a pivotal role for the functionality of muscle spindles and the application of glutamate leads to an augmentation in the spindle sensitivity.^3^^,^^4^^,^^26^ The presence of VGLUT1 in muscle spindles and Golgi tendon organs of pig EOMs demonstrates a neuromolecular similarity between proprioceptors of EOMs and skeletal muscle. It is therefore likely, that analog to skeletal muscle spindles, glutamate plays a significant role in the function of EOM proprioceptors. In summary, immunolabeling with anti-ChAT and anti-VGLUT1 demonstrates the presence of distinct neurotransmitters in palisade endings and classical proprioceptors of pig EOMs, suggesting divergent functionality among these structures.

It has been demonstrated in murine skeletal muscle spindles that annulospiral sensory endings are cholinergic and associated with acetylcholine receptors, as evidenced by the presence of ChAT and α-bungarotoxin.^41^ The blocking of acetylcholine receptors increases the sensitivity of annulospiral sensory endings.^42^ Annulospiral sensory endings coupled with acetylcholine receptors, stand in sharp contrast to the findings of the present study. Specifically, annulospiral sensory endings in the pig EOM spindles are non-cholinergic and lack acetylcholine receptors, as demonstrated by the absence of ChAT and α-bungarotoxin. At the moment, these discrepancies between skeletal and EOM spindles remain unexplained, underscoring the necessity for further investigation.

Despite the evident differences between classical proprioceptors and palisade endings, data in the literature^27^^,^^43^^,^^44^ indicate that these organs also share molecular characteristics. Specifically, calcium-binding protein calretinin has been identified in some palisade endings of the medial and inferior rectus muscles of primates,^27^^,^^44^ as well as in human EOM spindles^44^ and skeletal muscle spindles.^43^ The presence of synaptophysin, synaptobrevin, and complexin in classical proprioceptors and palisade endings of pig EOMs uncovered additional molecular parallels between these structures. Synaptophysin, synaptobrevin, and complexin are key players in the process of neurotransmitter release.^29^^,^^45^ Their broad distribution indicates that the proteins of the exocytotic machinery are conserved in classical proprioceptors and palisade endings, suggesting a relationship with other parameters rather than their sensory or motor properties.

### Functional Considerations

Our findings show that muscle spindles and Golgi tendon organs in EOMs and their equivalents in skeletal muscles have molecular features in common. It is therefore most likely that EOM proprioceptors possess an analogous function and register changes in muscle length and tension. Support that EOM spindles respond to changes in muscle length came from an older study.^46^ Specifically, following eye muscle stretching afferent signals, resembling those of muscle spindles were recorded in the sensory trigeminal ganglion of goats and sheep.^46^ The number of muscle spindles and Golgi tendon organs is extremely high in the EOMs of even-toed ungulate. A total of 181 muscle spindles were counted in the superior rectus of sheep,^47^ and between 148 and 310 muscle spindles were in pig EOMs,^48^ depending on the specific muscle. The number of Golgi tendon organs in pig EOMs ranged from 50 to 150. The high amount of proprioceptors suggests that the brain receives minute feedback from the EOMs to contribute to the sense of eye position. A study in humans suggests that proprioceptive signals from EOM muscle spindles reach the anterior parietal cortex.^49^ The pathways through which these signals reach the brain remain to be elucidated.

Palisade endings are located at the myotendinous junction, a position analogous to that of Golgi tendon organs, which theoretically allows them to register the contraction of muscle fibers. However, our study demonstrates that palisade endings exhibit structural and neurotransmitter characteristics that are not compatible with proprioceptive function, putting into question whether palisade endings can substitute classical proprioceptors in those animals where they are missing.

## Supplementary Material

Supplement 1
