# Supplementary material for: Classical Proprioceptors and Palisade Endings Have Distinct Molecular Profiles in Pig Eye Muscles
Source: Invest Ophthalmol Vis Sci. 2026 Mar 2;67(3):1. doi: 10.1167/iovs.67.3.1 (PMC12967111; doi:10.1167/iovs.67.3.1)
Supplement: Supplement 1 [file iovs-67-3-1_s001.docx]

**
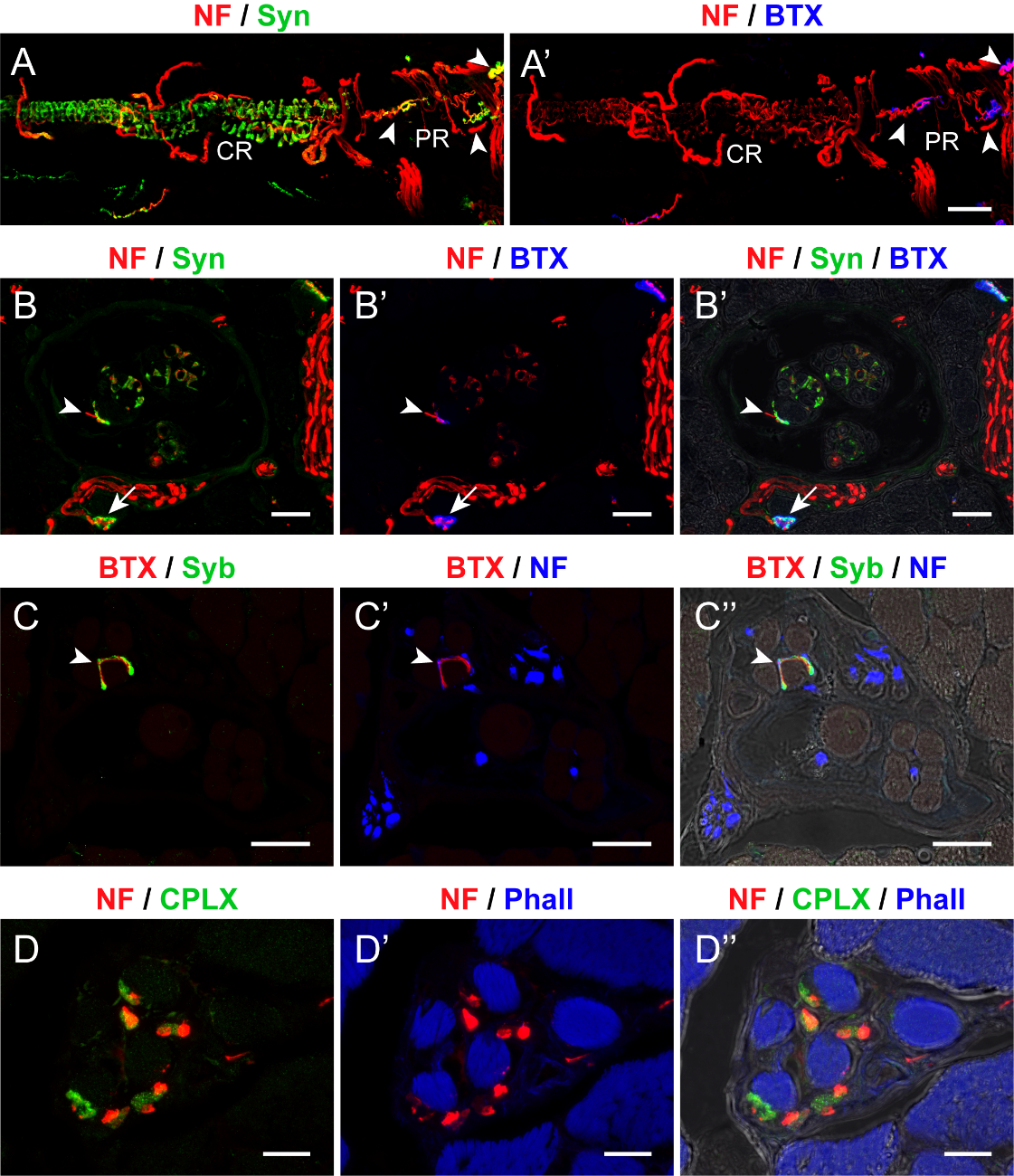
**

**Supplementary figure 1. Presynaptic molecules in motor terminals of muscle spindles.** (**A – A’**) The image in figure **2C** is shown in a split channel configuration, specifically in neurofilament (NF, red) / synaptophysin (Syn, green) (**A**) and in NF / α-bungarotoxin (BTX, blue) (**A**’). Motor terminals (white arrowheads) express synaptophysin (**A**) and α-bungarotoxin (**A’**). Central region (CR), paraequatorial region (PR). (**B – B”**) The image in figure **2D** is shown in a split channel configuration, specifically in neurofilament (NF, red) / synaptophysin (Syn, green) (**B**), NF / α-bungarotoxin (BTX, blue) (**B**’), and in the overlay along with light transmission (**B”**). A motor terminal within the spindle’s paraequatorial region (white arrowhead) and a motor terminal outside the spindle (white arrow) express synaptophysin (**B**) and α-bungarotoxin (**B’**). (**C – C”**, **D – D”**) Cross-sections of the muscle spindle’s polar region, labelled with anti-neurofilament (NF, blue) / anti-synaptobrevin (Syb, green) / α-bungarotoxin (BTX, red) in **C – C”**, and anti-neurofilament (NF, blue) / anti-complexin (CPLX, green) / phalloidin (Phall, blue) in **D – D”**. Motor terminals within the spindle express synaptobrevin and α-bungarotoxin (white arrowhead in **C – C”**) and complexin (**D – D’’**). Scale bars: 50 μm in **A – A’**; 25 μm in **B – B’’**; 15 μm in **C – C’’**; 25 μm in **D – D’’**.
